# The Influence of the Accelerated Aging Conditions on the Properties of Polyolefin Geogrids Used for Landfill Slope Reinforcement

**DOI:** 10.3390/polym12091874

**Published:** 2020-08-20

**Authors:** Agnieszka Kiersnowska, Wojciech Fabianowski, Eugeniusz Koda

**Affiliations:** 1 Institute of Civil Engineering, Warsaw University of Life Sciences—SGGW, 159 Nowoursynowska Str., 02-787 Warsaw, Poland; eugeniusz_koda@sggw.edu.pl; 2 Faculty of Chemistry, Warsaw University of Technology, 3 Noakowskiego Str., 00-661 Warsaw, Poland; wofab@ch.pw.edu.pl

**Keywords:** polyolefin, geosynthetics, HDPE, landfill, degradation, accelerated aging tests, decrease mechanical properties

## Abstract

Polyolefin geosynthetics are susceptible to oxidative degradation, which in turn leads to diminished mechanical properties in geotechnical constructions. When using these materials, it is extremely important to determine their durability over time in particularly aggressive conditions. In order to prolong the life of a geosynthetic material, antioxidants are added during the manufacturing process. The function of antioxidants is to prevent polymer oxidation reaction in time. As the antioxidant content is depleted, the polymer becomes less protected towards oxidative attacks. This article describes the aging process of uniaxial (high density polyethylene) HDPE geogrids under the influence of chemical and environmental factors. Evaluations of accelerated aging test of the uniaxial HDPE geogrids were incubated in simulated landfill conditions for a period of 12 months. Three temperatures (25 °C, 45 °C, and 75 °C) were selected for carrying out the aging experiments in aqueous solutions mimicking landfill conditions. The changes observed by differential scanning calorimetry (DSC), Fourier transform infrared spectroscopy (FTIR) and melt flow index (MFI) correlate with the mechanical properties of the aged geogrid. No significant changes in the FTIR and MFI were observed over the 12 months of accelerated aging tests at none of the three different temperatures. The oxidation induction time (OIT) test showed no antioxidant remaining in the geogrid following eight months of aging test at 75 °C. No significant changes in the influence of accelerated aging tests on the average relative elongation at 25 °C and 45 °C of the tested material were observed. Accelerated aging tests at 75 °C showed that the mean elongation of 12.12% for the sample not subjected to accelerated aging tests (new sample) increased to 19.32% (after 12 months of incubation).

## 1. Introduction

In the geosynthetic industry, polyolefin refers to synthetic products consisting of at least 85% by weight of polyethylene (PE) or polypropylene (PP) [1,2]. The use of polyolefin geosynthetics has been constantly increasing in different areas of civil engineering applications. HDPE (high density polyethylene) geogrids have been used as reinforcement materials in slopes, walls, foundations, and base courses for about five decades. First reinforced soil structures in the world were built in France in 1970 [3,4]. In the United States, structures of this type were built since 1974 [5] and in Poland, in engineering practice, they are used for about 30 years [6].

HDPE geogrids have found wide applications in the design and construction of landfills. The latter application has been triggered by the economic and technical advantages that geosynthetics offer in relation to more traditional materials [1,7,8]. The service life of a structure with a geosynthetic material largely depends on its durability [2]. The required service lives (about 100 years) of many structures significantly challenge the longevity of the polyolefin geosynthetics. Because of this, the properties of geosynthetics are always of interest to civil engineers [9]. The aging process of geosynthetic materials can be described in terms of both physical and chemical deterioration [9,10,11,12,13]. In physical aging, the material attempts to establish an equilibrium from its manufactured non-equilibrium state. Here, no primary (covalent) bonds are broken, however for semi-crystalline polymers such as HDPE, an increase in the material crystallinity occurs. Physical aging can be represented as mass transfer with the environment surrounding the material i.e., extraction of additive, absorption of solvent, and as modifications of the organization of the internal chain in the material i.e., chain orientation and crystallinity [14]. In chemical aging, bond scission in the backbone leads to the formation of macromolecules, intermolecular cross-linking, and various other chemical reactions [12]. The geosynthetics may be simultaneously subject to several types of degradation processes during their service life. Different degradation mechanisms may have synergistic effects that could accelerate the overall process.

Polyolefin materials undergo the adverse effects of an oxidation reactions, that can occur at any stage in the life cycle of the polymer. During both processing and service, polyolefins are susceptible to oxidative degradation, that eventually leads to a reduction in engineering properties caused by the polymer chain scission. The sequential steps of the oxidation chain reaction include initiation, propagation, chain branching and finally the termination reactions [15,16,17]. In order to delay the onset of the oxidation process, antioxidants are typically added to the base material at 0.5 to 1% level. Antioxidants are depleted by chemical reactions, thereby preventing the oxidative degradation of polymers and reduce the physical losses by volatilization or extraction [16].

**Figure 1:**
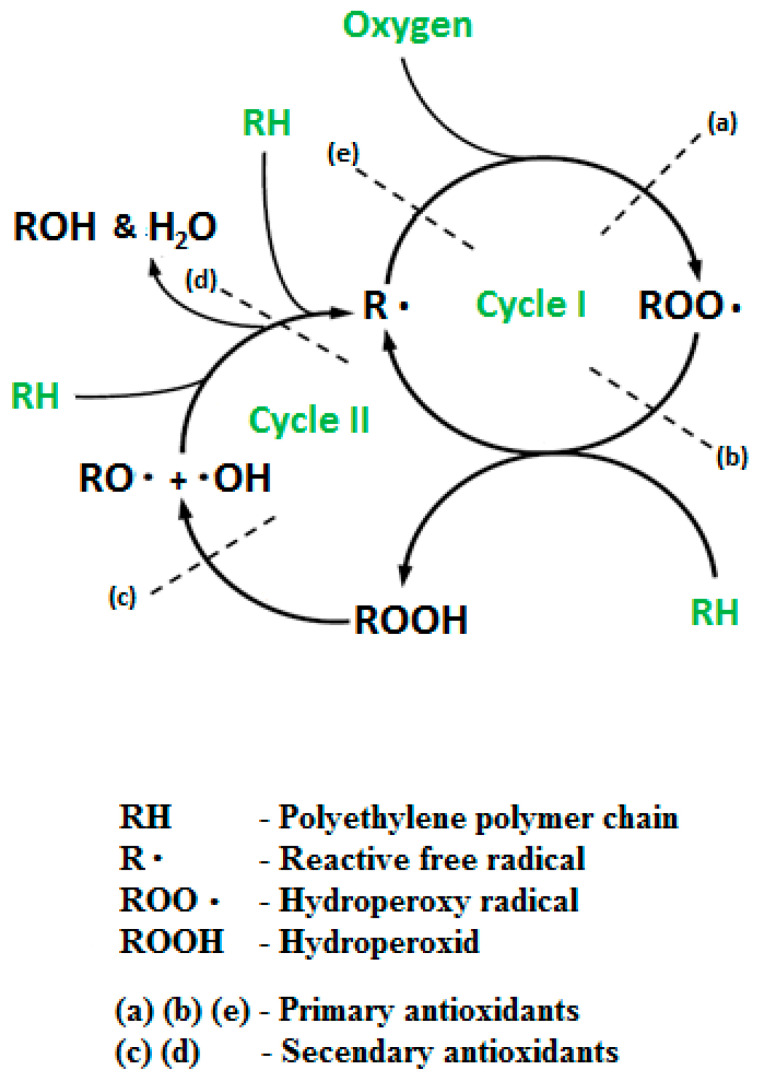
The oxidation cycle in polyethylene (modified from Rowe and Sangam, 2002).

The oxidation mechanism of polyethylene can be presented as two cycle process (Figure 1). The cycle I a chain reaction of alkyl/alkylperoxyl, while the cycle II is the formation of new radicals by chain reaction (homolysis of hydroperoxides). The good performance of antioxidants depends on the quantity, their type, and the temperature. The primary antioxidants work to prevent free radical formation, and the secondary antioxidants work to decrease the formation of active hydroperoxides in inactive alcohols [12,18,19].

The oxidative degradation of polyethylene can be divided into three stages:

Stage I covers the time of depletion of antioxidants caused by their consumption as a result of a chemical reaction with oxygen.

Stage II includes the induction time needed for the oxidation degradation of the polymer.

Stage III involves the actual degradation of the polymer leading to the decrease of its mechanical properties [20].

Even in a relatively inert environment, synthetic materials deteriorate over time when exposed to mechanical stress. However, given the conditions prevailing at the landfills, the geosynthetics are exposed to the particularly chemically aggressive substances. In addition, either soil or liquid in direct contact with olefine geosynthetics can have a significant effect on the oxidation rate. The presence of heavy metals accelerates the oxidation reaction rate of olefine polymers. The exposure condition that may significantly enhance the oxidation of a geosynthetic is the presence of transition metals such as Fe, Zn, Mn, and Cu in leachate. These metals break down hydroperoxides (redox reactions) and create additional free radicals [21].

Temperature also affects the physical and mechanical properties of the geosynthetic. In landfills, the temperature may exceed 70 °C [22,23]. However, the average temperature varies depending on the location and the season. There, the elevated temperatures lead to the thermo-oxidation degradation. An important factor that affects the oxidation rate is the availability and the abundance of oxygen (the concentration of available oxygen is an essential component of any oxidation reaction).

This paper focuses on the durability of HDPE uniaxial geogrids used for the reinforcement of landfills slope. Tests of accelerated aging process of the HDPE uniaxial geogrids were provided in simulated landfill conditions over a period of 12 months. For this study, three temperatures were selected (25 °C, 45 °C, and 75 °C). Heavy metals in leachate catalyze decomposition of hydro peroxide to generate free radicals and deplete the antioxidants [21,24]. Surfactants in the leachate increase the wetting ability of the geosynthetics [25]. The surfactants are common in landfills, originating predominantly from laundry detergents and bath soaps in the waste stream [26,27].

The accelerated aging tests of geosynthetics are very important for the reclamation of old municipal landfills, often with high slope inclinations, where improvement of stability conditions is necessary for their final development. In Poland, about 1000 such facilities have been closed in the last 10 years.

## 2. Materials and Methods

**Figure 2:**
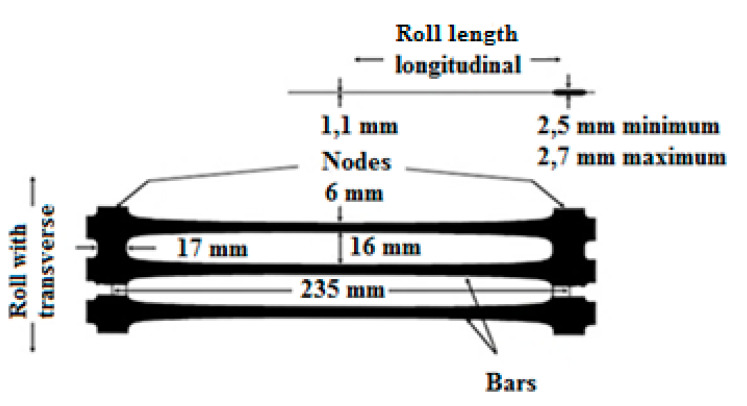
Dimensions of uniaxial geogrid [28].

The geogrid production process begins with extrusion of a HDPE sheet perforated in a regular pattern. In controlled heating conditions, the sheet is then stretched out at a temperature above glass transition but well below the melting point. This process increases the size of the punched holes and orients the polymer molecules in the direction of stretch. The process is performed to increase the tensile strength and tensile stiffness of the polymer [28]. The structure of the geogrid is shown in Figure 2 and the corresponding main properties are listed in Table 1.

### 2.1. Accelerated Ageing Test

The accelerated laboratory tests involve immersion of the HDPE uniaxial geogrid coupons in a synthetic leachate. The accelerated aging tests were planned based on the literature data that described operating conditions prevailing in landfills in Poland [33]. In particular Sangam and Rowe (2002) investigated antioxidant depletion in a HDPE geomembrane exposed to leachate, water and air at different temperatures (22 °C, 40 °C, 55 °C, 70 °C, and 85 °C). The synthetic leachate contained the surfactant Igepal CA-720, e.g., acetic acid, butyric acid, propionic acid, and trace metals.

**Figure 3:**
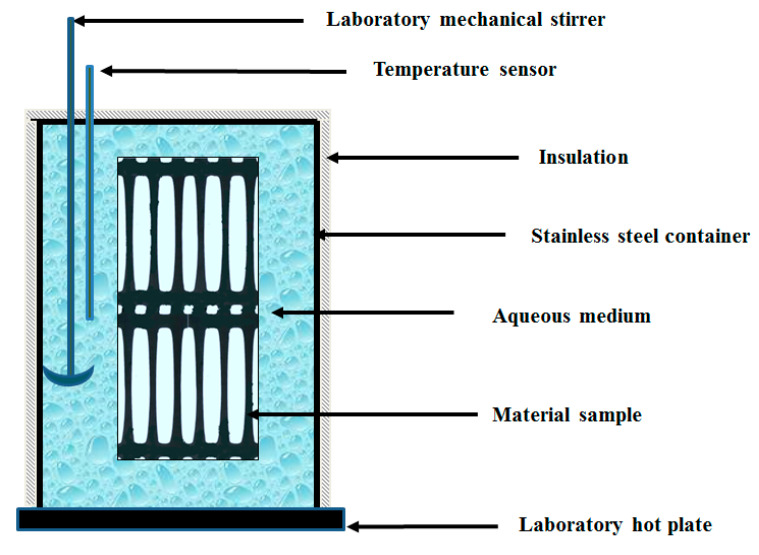
Apparatus employed for accelerated aging evaluations.

For the purpose of this study aqueous solutions of various heavy metal compositions were selected. Several temperatures were likewise employed. The aging tests were carried out at 25 °C, 45 °C, and 75 °C, covering the range of temperatures found in these facilities. The composition of the solution simulating the environment is presented in Table 2 and Table 3. The values were taken from the study [18] and modified to the conditions prevailing at landfills in Poland. In this study, we employed Triton X-100 representative surfactant, generally used in detergents and cosmetics. Geogrids coupons (540 by 240 mm in size) were placed in 50-L stainless steel containers equipped with a mechanical stirrer and a temperature sensor. The scheme of the apparatus for accelerated aging test is shown on a diagram in Figure 3. Distilled water was used in making leachates for the aging tests programs. The coupons were separated using glass rods.

### 2.2. Oxidation Induction Time (OIT)

**Figure 4:**
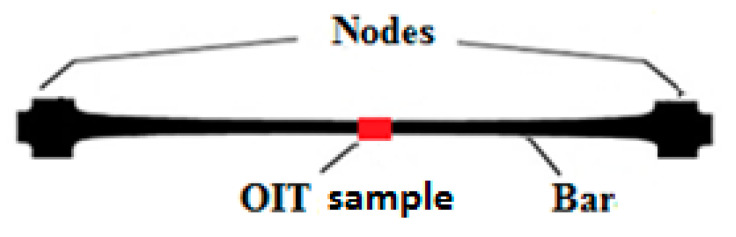
Sampling locations of oxidation induction time OIT test samples.

OIT test is performed by using a differential scanning calorimetry (DSC) to measure the thermal stabilization of the test sample. The time elapsed between the melting process and the onset of decomposition in isothermal conditions is measured. The OIT can be used to assess the amount of antioxidant present in the geosynthetics [12,16]. The Termo Analyses DSC Q 20 series (TA Instruments, New Castle, DE, USA) was used in this study. Standard OIT tests were conducted in accordance with the PN EN ISO 11357-6 procedure. A 3 to 8 mg sample was heated to 200 °C at a rate of 20 °C/min in a nitrogen atmosphere. After 200 °C had been reached, the sample was maintained in an isothermal state for 5 min. Then, the purge gas was changed from nitrogen to oxygen, and the change in Enthalpy was recorded. The test was terminated when an exothermal peak was detected. OIT samples were taken from one (central) part as indicated in Figure 4, three replicates were tested in each incubated interval.

### 2.3. Melt Flow Index (MFI)

MFI is a method to assess the molecular weight of polymeric materials. It is commonly used as an index of molecular weight in chemical compatibility studies of geosynthetics [16]. The chain scission reactions, one of the most important outcomes of degradation, produce smaller polymer molecules [15]. During the aging of the polymer, a cross-linking or chain scission reaction can occur in the polymer chain [16]. The cross-linking reaction leads to a decrease in the MFI value, while the reaction to chain scission results increase in MFI value. This change in molecular size is reflected in a higher MFI. The MFI evaluation was carried out on a Tinus Olsen MP 1200 (Tinus Olsen, Horsham, PA, USA. The test measures the amount of molten polymer extruded through an orifice of a specified diameter at 190 °C in g/10 min under a constant load of 2.16 kg in accordance with the PN-EN ISO 1133:2005 procedure.

### 2.4. Fourier Transform Infrared Spectroscopy (FTIR)

FTIR is a spectroscopic method used to detect structural changes in polymeric materials at a molecular level. FTIR allows to determine the type of functional groups present in the structure of the test compound, which allows to specify the qualitative composition of the sample. Changes observed in the spectrum can be used as indicators of geosynthetics degradation [34]. The FTIR were collected on a Thermo Scientific, Nicolet 6700 (Thermo Scientific, Waltham, MA, USA) with an adapter for measurements in reflective mode. The examined absorption covered the range of 400 to 4000 cm^−1^ at a resolution of 4 cm^−1^. Each spectrum consisted of 32 scans.

### 2.5. Tensile Strength Tests

In the case where the geosynthetics function as reinforcement their tensile strength, the elongation at maximum load are crucial for the assessment of the product stability, since the action of elevated or reduced temperature and humidity changes their properties. The tensile properties of new and aged geogrid samples were evaluated an Instron Universal Testing Machine (Instron, Noworwood, MA, USA) according to the PN EN ISO 10319 testing method. For each test five specimens were used. The monotonic tensile tests performed at a rate of strain equal to 20%/min. Since the specimens were approximately 470 mm long, the rate of the machine was set at 94 mm/min.

## 3. Results and Discussion

### 3.1. Oxidation Induction Time

**Figure 5:**
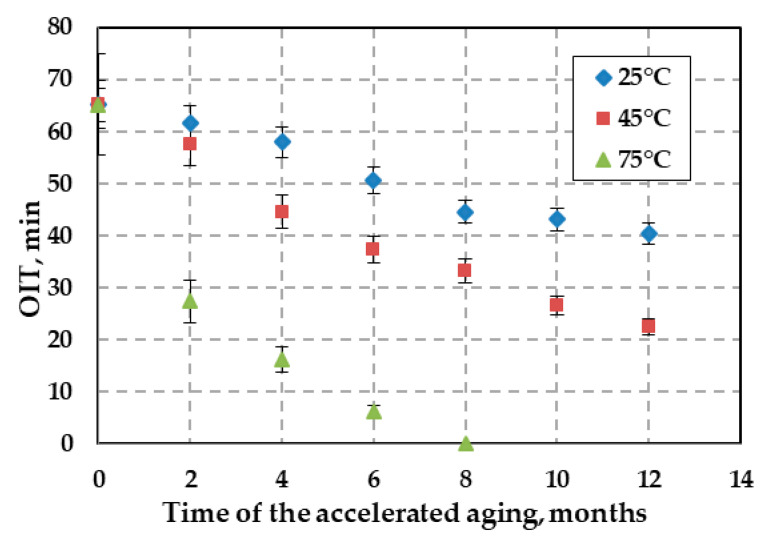
Variation in oxidation induction time (OIT) of the high density polyethylene (HDPE) geogrid sample with time in leachate at three temperatures: 25 °C, 45 °C, and 75 °C.

OIT as a function of exposure time for the HDPE geogrid is shown in Figure 5. For a sample aged over 12 months at 25 °C, an OIT of 40.33 min was obtained. The antioxidant concentration in relation to the reference sample decreased by 38%. For a sample aged over 12 months at 45 °C, an OIT of 22.18 min was obtained. The antioxidant concentration in relation to the reference sample decreased by about 66%. The OIT decreased faster for the higher temperatures. For example, the OIT of the antioxidant from the geogrid subject to accelerated aging tests at 75 °C was the fastest. OIT tests for a sample after eight months of aging (75 °C) showed no antioxidant remaining. Thus, this ended the Stage I of degradation of the geogrid. The trend of accelerating the process of antioxidant depletion with increasing temperature is evident, especially at a temperature of 75 °C, which may occur inside the mass of the MSW landfill.

### 3.2. Melt Flow Index

**Figure 6:**
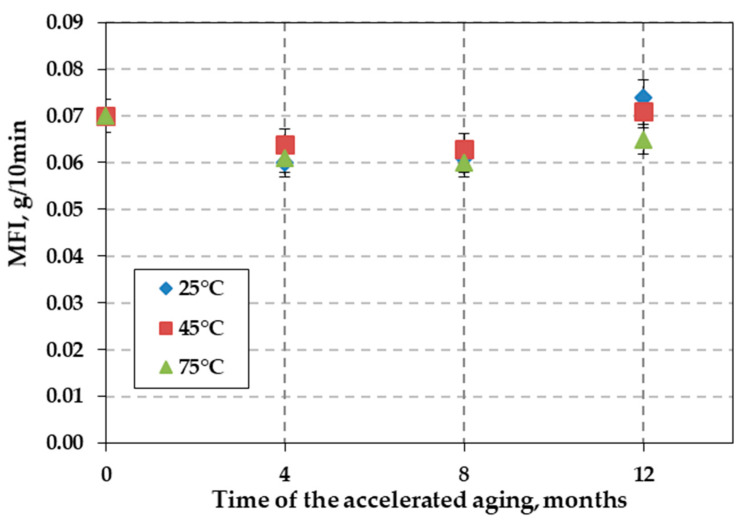
Variation in melt flow index (MFI) of the HDPE geogrid sample with time in leachate at three temperatures: 25 °C, 45 °C, and 75 °C.

In this study, no significant changes in the MFI were observed over the 12 months of accelerated aging tests at 25 °C and 45 °C (Figure 6). A decrease in MFI values was noted after four and eight months for samples incubated at all temperatures. A decrease in the MFI value indicates a cross-linking reaction. However, MFI increased for samples at all temperatures after 12 months of incubation. The obtained MFI values ranged from 0.061 to 0.074 g/10 min. Observed raise in the MFI value for over 20% is significant and indicates that decomposition of the HDPE aged at the highest temperature started to proceed. Even the biggest change can be observed for the samples aged at the 25 °C, but this was not confirmed by the analysis of the FTIR spectra. The ongoing analysis aimed to explain the observed results.

### 3.3. FTIR Spectroscopy

The high testing temperatures may induce morphological changes in the polymeric product. In turn, these affect the kinetics of the oxidative degradation that results in the formation of hydroxyls, carbonyl and carboxylic groups, ether, peroxides, and hydroperoxides. FTIR spectroscopy was used to identify the oxygen bearing groups formed during in the leachate degradation processes.

**Figure 7:**
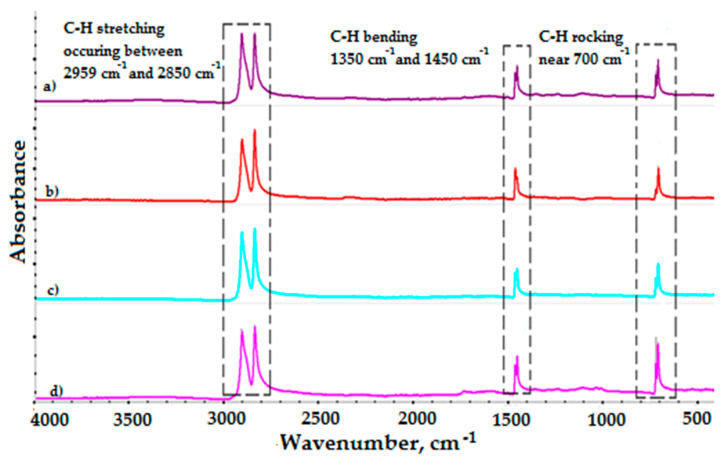
Fourier transform infrared spectroscopy (FTIR spectra) of: (**a**) the new HDPE geogrid sample, (**b**) the aged HDPE geogrid sample in leachate for 12 months at 25 °C, (**c**) the aged HDPE geogrid sample in leachate for 12 months at 45 °C, and (**d**) the aged HDPE geogrid sample in leachate for 12 months at 75 °C.

Figure 7 shows the FTIR spectra of new and aged HDPE uniaxial geogrids aged in leachates for 12 months at three different temperatures at 25 °C, 45 °C, and 75 °C, respectively. For both materials (geogrid new and aged), three absorptions bands can be observed. These are: the C-H stretching mode between 2950 and 2850 cm^−1^, the C-H bending mode between 1350 and 1450 cm^−1^ and the C-H rocking vibration near 700 cm^−1^. Significant absorption bands originating from the oxidation of the HDPE matrix were not observed. The small absorbance of carbonyl group in spectra (1730 cm^−1^) of HDPE sample aged at 75 °C indicate proceeding degradation of polyethylene chain. Nevertheless, the absence of major changes in the spectra suggests that exposure to solution at 75 °C did not significantly change the bulk polymer structure over 12 months.

### 3.4. Tensile Strength Tests

**Figure 8:**
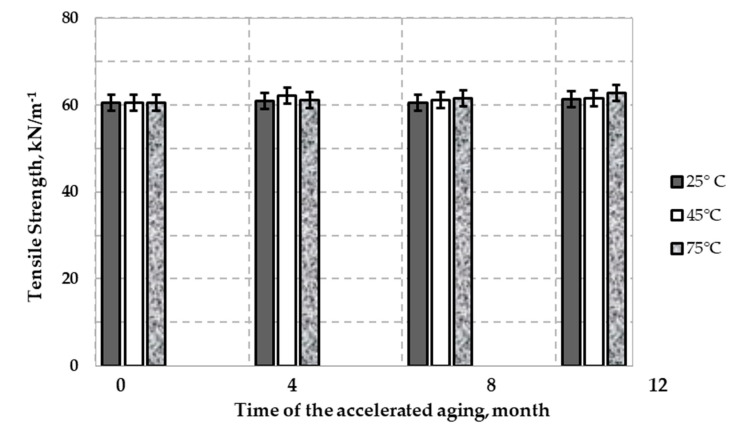
Variation of tensile strength (T)HDPE geogrid sample with time in leachate at three temperatures: 25 °C, 45 °C, and 75 °C.

Figure 8 shows the dependence of the average tensile strength (T) on the time of aging samples of HDPE samples of uniaxial geogrid. The average tensile strength of the new HDPE uniaxial geogrid samples was measured to be 60.56 kN/m. The mean values of tensile strength for samples incubated at 25 °C were measured in the range of 61.00 kN/m (after four months of incubation) to 61.35 kN/m (after 12 months of incubation). The mean values of tensile strength for samples incubated at 45 °C were measured in the range of 62.20 kN/m (after four months of incubation) to 61.55 kN/m (after 12 months of incubation). For samples incubated at 75 °C, mean T values were measured in the range of 61.06 kN/m (after two months of incubation) to 62.75 kN/m (after 12 months of incubation). After 12 months of accelerated aging tests at individual temperatures, no statistically significant changes affecting the average tensile strength were found by the wide-width-tensile test.

**Figure 9:**
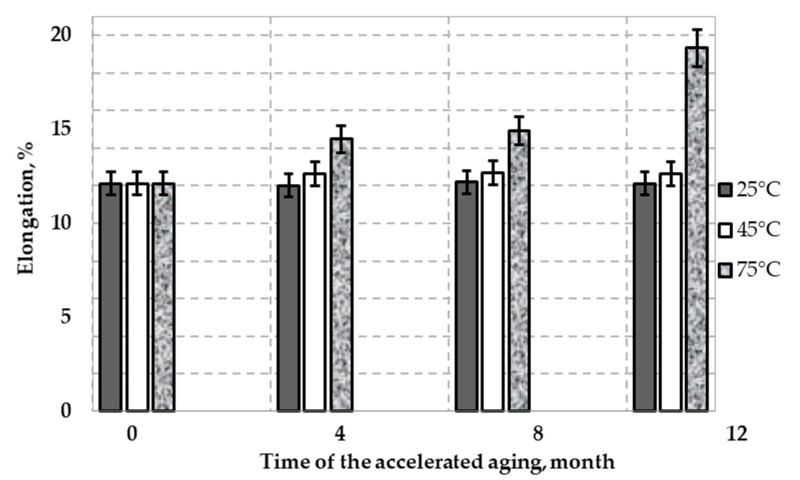
Variation of relative elongation (ε) HDPE geogrid sample with time in leachate at three temperatures: 25 °C, 45 °C, and 75 °C.

No significant changes in the influence of accelerated aging tests on the average relative elongation at 25 °C and 45 °C of the tested material were observed (Figure 9.). Accelerated aging tests at 75 °C showed that the mean elongation of 12.12% for the sample not subject to accelerated aging tests (new sample) increased to 19.32% (after 12 months of incubation). The ongoing analyses aimed at explaining the observed results. This phenomenon indicates that high temperature incubation was not advisable for this uniaxial geogrid. Further increase in temperature may cause attainment values that can significantly affect the reduction reinforcement functions of the materials.

Studies confirm that in an aggressive environment of landfills, accelerated material aging and reduction of mechanical parameters may occur. This is particularly important when using the uniaxial geogrid to reinforce the stability of slopes of embankment type landfills [35], which in extreme conditions may lead to landslides and other threats to surroundings areas.

## 4. Conclusions

Tests of accelerated aging of the HDPE uniaxial geogrid were provided in incubated in simulated landfill conditions for a period of 12 months. More detailed changes in the mechanical and physicochemical properties were noted as follows:

(1) The rate of depletion of the antioxidant depends on the temperature (OIT test). The results of the OIT test showed no antioxidant remaining in the geogrid following eight months of test aging at 75 °C.
(2) No significant changes in the MFI were observed over the 12 months of accelerated aging tests at 25 °C and 45 °C. However, observed raise in the MFI value for over 20% is significant and indicates that decomposition of the HDPE aged at the highest temperature.
(3) FTIR spectra of the topmost layer of the new and aged samples, respectively, showed no differences at 25 °C and 45 °C. There were no significant changes compared to the topmost layer of the new samples. Small absorbance of carbonyl group in spectra (1730 cm^−1^) of HDPE sample aged at 75 °C indicate proceeding degradation of polyethylene chain.
(4) Results of the MFI and FTIR tests showed that exposure to solution had little effect on the polymer properties. No consistent changes in the polymer structure were observed after 12 months.
(5) Accelerated aging tests at 75 °C showed that the mean elongation of 12.12% for the sample not subjected to accelerated aging tests (new sample) increased to 19.32% (after 12 months of incubation). This result indicates that a high temperature incubation was not recommended for this geogrid.
(6) Assessing the accelerated aging process of polyolefin geogrids used to reinforce landfill slopes is important to prevent landslides.

## Figures and Tables

**Table 1:** The main engineering properties of the uniaxial geogrid.

Tests	Parameters	Samples Mean Value	Specific Standard
Mechanical	Tensile strength (kN/m)	60.5	PN-EN ISO 10319 [29]
	Strain at Maximum Load (%)	12.1	
Resin	Melt Flow Index (g/10 min)	0.07	PN-EN ISO 1133-1 [30]
	Oxidation induction time (min)	65	PN-EN ISO 11357-6 [31]
	Glass transition temperature (°C)	−100	Biron 2018 [32]
	Melting temperature (°C)	130	

**Table 2:** The composition of synthetic leachate used in the laboratory.

Component	Concentration, mL/L (Except Where Noted)
Surfactant, Triton X-100	5
Solution of heavy metals ^a^	1
pH (adjusted)	6

^a^ See Table 3 for the compositions.

**Table 3:** The composition of the solution of heavy metals.

Component	Concentration, mg/L
Ferrous Sulfate (FeSO_4_·7H_2_O)	4480
Zinc Sulfate Heptahydrate (ZnSO_4_·7H_2_O)	360
Cupric Sulfate Pentahydrate (CuSO_4_·5H_2_O)	40
Aluminum Sulphate 16-Hydrate (Al_2_(SO_4_)_3_·16H_2_O)	30
Manganous Sulfate 4-Hydrate (MnSO_4_·4H_2_O)	60
Nickel Sulfate 6-Hydrate (NiSO_4_·6H_2_O)	50
